# Adding Recognition Discriminability Index to the Delayed Recall Is Useful to Predict Conversion from Mild Cognitive Impairment to Alzheimer's Disease in the Alzheimer's Disease Neuroimaging Initiative

**DOI:** 10.3389/fnagi.2017.00046

**Published:** 2017-03-10

**Authors:** María J. Russo, Jorge Campos, Silvia Vázquez, Gustavo Sevlever, Ricardo F. Allegri, the Alzheimer's Disease Neuroimaging Initiative, Michael W. Weiner

**Affiliations:** Author Affiliations: UC San Francisco; University of Southern California; UC San Francisco University of Southern California Mayo Clinic, Rochester Mayo Clinic, Rochester; UC Berkeley; U Pennsylvania; USC; UC Davis; Brigham and Women's Hospital/Harvard Medical School Indiana University Washington University St. Louis University of Pennsylvania; Prevent Alzheimer's Disease 2020 (Chair) Siemens; Alzheimer's Association University of Pittsburgh Washington University St. Louis Cornell University; Albert Einstein College of Medicine of Yeshiva University; AD Drug Discovery Foundation; Acumen Pharmaceuticals; Washington University St. Louis; Northwestern University; National Institute of Mental Health; Brown University; Eli Lilly (Chair); BWH/HMS (Chair); University of Washington (Chair); Mayo Clinic, Rochester (Core PI) University of Southern California; UC San Diego; UC San Diego; UC San Diego; UC San Diego; UC San Diego; UC San Diego; UC San Diego; UC San Diego; UC San Diego; UC Davis (Core PI); UC Davis; UC San Diego; Mayo Clinic, Rochester (Core PI); Mayo Clinic, Rochester; University of London; UCLA School of Medicine; UCSF MRI; UC Davis; Mayo Clinic; Mayo Clinic; Mayo Clinic; Mayo Clinic; Mayo Clinic; Mayo Clinic; Mayo Clinic; UC Berkeley (Core PI); University of Michigan; University of Utah; Banner Alzheimer's Institute; Banner Alzheimer's Institute; University of Pittsburgh; UC Berkeley; Washington University St. Louis; Washington University St. Louis; Washington University St. Louis; Washington University St. Louis; UPenn School of Medicine; UPenn School of Medicine; UPenn School of Medicine; UPenn School of Medicine; UPenn School of Medicine; USC (Core PI); USC; USC; Indiana University; Indiana University; UC Irvine; Indiana University; Indiana University; Indiana University; Indiana University; UC San Francisco; UC San Diego; Prevent Alzheimer's Disease 2020; UC San Diego; National Institute on Aging; UC San Francisco; Brown University; National Institute of Mental Health; Cornell University; Johns Hopkins University; Richard Frank Consulting; Prevent Alzheimer's Disease 2020; National Institute on Aging; Oregon Health & Science University; University of Southern California; University of California - San Diego; University of Michigan; Mayo Clinic, Rochester; Baylor College of Medicine; Columbia University Medical Center; Washington University, St. Louis; University of Alabama - Birmingham; Mount Sinai School of Medicine; Rush University Medical Center; Wien Center; Johns Hopkins University; New York University; Duke University Medical Center; University of Pennsylvania; University of Kentucky; University of Pittsburgh; University of Rochester Medical Center; University of California, Irvine; University of Texas Southwestern Medical School; Emory University; University of Kansas, Medical Center; University of California, Los Angeles; Mayo Clinic, Jacksonville; Indiana University; Yale University School of Medicine; McGill Univ., Montreal-Jewish General Hospital; Sunnybrook Health Sciences, Ontario; U.B.C. Clinic for AD & Related Disorders; Cognitive Neurology - St. Joseph's, Ontario; Cleveland Clinic Lou Ruvo Center for Brain Health; Northwestern University; Premiere Research Inst (Palm Beach Neurology); Georgetown University Medical Center; Brigham and Women's Hospital; Stanford University; Banner Sun Health Research Institute; Boston University; Howard University; Case Western Reserve University; University of California, Davis - Sacramento; Neurological Care of CNY; Parkwood Hospital; University of Wisconsin; University of California, Irvine - BIC; Banner Alzheimer's Institute; Dent Neurologic Institute; Ohio State University; Albany Medical College; Hartford Hospital, Olin Neuropsychiatry Research Center; Dartmouth-Hitchcock Medical Center; Wake Forest University Health Sciences; Rhode Island Hospital; Butler Hospital; UC San Francisco; Medical University South Carolina; St. Joseph's Health Care; Nathan Kline Institute; University of Iowa College of Medicine; Cornell University; University of South Florida: USF Health Byrd Alzheimer's Institute; University of California, San Francisco; University of Southern California; UC San Francisco; University of Southern California; Mayo Clinic, Rochester; Brigham and Women's Hospital/ Harvard Medical School; UC Davis; Mayo Clinic, Rochester; UC Berkeley; Washington University St. Louis; Indiana University; Perelman School of Medicine, UPenn; USC; Perelman School of Medicine, University of Pennsylvania; UC San Francisco; Rehabilitation Institute of Chicago, Feinberg School of Medicine, Northwestern University; BWH/HMS (Chair); University of Washington (Chair); Core PI; Mayo Clinic, Rochester (Core PI); University of Southern California; UC San Diego; UC San Diego; UC San Diego; UC San Diego; UC San Diego; UC San Diego; UC San Diego; UC San Francisco; UC San Francisco; UC San Francisco; UC Davis (Core PI); UC San Diego; Mayo Clinic, Rochester (Core PI); Mayo Clinic, Rochester; Mayo Clinic; Mayo Clinic; Mayo Clinic; Mayo Clinic; Mayo Clinic; UC Berkeley (Core PI); University of Michigan; University of Utah; Banner Alzheimer's Institute; Banner Alzheimer's Institute; UC Berkeley; Washington University St. Louis; Washington University St. Louis; Washington University St. Louis; Perelman School of Medicine, UPenn; Perelman School of Medicine, UPenn; Perelman School of Medicine, UPenn; Perelman School of Medicine, UPenn; Perelman School of Medicine, UPenn; USC (Core PI); USC; USC; Indiana University; Indiana University; UC Irvine; Indiana University; Indiana University; Indiana University; Indiana University; UC San Francisco; Department of Defense (retired); University of Southern California; University of California, San Diego; Columbia University Medical Center; Rush University Medical Center; Wien Center; Duke University Medical Center; University of Rochester Medical Center; University of California, Irvine; Medical University South Carolina; Premiere Research Inst (Palm Beach Neurology); University of California, San Francisco; Georgetown University Medical Center; Brigham and Women's Hospital; Banner Sun Health Research Institute; Howard University; University of Wisconsin; University of Washington; Stanford University; Cornell University; Aging and Memory Center, Instituto de Investigaciones Neurológicas Raúl Carrea, Fundación Para la Lucha Contra las Enfermedades Neurológicas de la Infancia (FLENI)Buenos Aires, Argentina

**Keywords:** disease progression, memory, recognition discriminability, mild cognitive impairment, Alzheimer's disease, signal detection theory

## Abstract

**Background:** Ongoing research is focusing on the identification of those individuals with mild cognitive impairment (MCI) who are most likely to convert to Alzheimer's disease (AD). We investigated whether recognition memory tasks in combination with delayed recall measure of episodic memory and CSF biomarkers can predict MCI to AD conversion at 24-month follow-up.

**Methods:** A total of 397 amnestic-MCI subjects from Alzheimer's disease Neuroimaging Initiative were included. Logistic regression modeling was done to assess the predictive value of all RAVLT measures, risk factors such as age, sex, education, APOE genotype, and CSF biomarkers for progression to AD. Estimating adjusted odds ratios was used to determine which variables would produce an optimal predictive model, and whether adding tests of interaction between the RAVLT Delayed Recall and recognition measures (traditional score and d-prime) would improve prediction of the conversion from a-MCI to AD.

**Results:** 112 (28.2%) subjects developed dementia and 285 (71.8%) subjects did not. Of the all included variables, CSF Aβ1-42 levels, RAVLT Delayed Recall, and the combination of RAVLT Delayed Recall and d-prime were predictive of progression to AD (χ^2^ = 38.23, *df* = 14, *p* < 0.001).

**Conclusions:** The combination of RAVLT Delayed Recall and d-prime measures may be predictor of conversion from MCI to AD in the ADNI cohort, especially in combination with amyloid biomarkers. A predictive model to help identify individuals at-risk for dementia should include not only traditional episodic memory measures (delayed recall or recognition), but also additional variables (d-prime) that allow the homogenization of the assessment procedures in the diagnosis of MCI.

## Introduction

Mild Cognitive Impairment (MCI) is a common condition defined as transitional state between normal cognition and dementia (Petersen et al., 2001). In general, subjects with MCI convert to dementia at an annual rate in the range of 10–15% (Farias et al., 2009). Predicting who among a group of MCI patients are more likely to further decline in cognition would be essential to ensure an early intervention and appropriate treatment as well as future preventive and treatment trials.

Of the MCI subtypes, patients with amnestic MCI (a-MCI) are at greatest risk. Poor delayed recall and recognition memory is a well-established pattern in the Alzheimer's disease (AD) literature, which is considered to reflect deficits in storage caused by deficient consolidation of new memory traces (Weintraub et al., 2012). Abundant evidence indicates delayed recall scores in word-list learning tasks such as the Rey Auditory Verbal Learning Test (RAVLT) (Rey, 1941, 1964) are perhaps the most challenging and accurate measures of episodic memory when used to accurately predict diagnostic conversion to AD (Estévez-González et al., 2003; Maruff et al., 2004; Griffith et al., 2006). It should be noted that other memory tests have shown predictive validity for clinical progression from MCI to AD, including Free and Cued Selective Reminding Test (FCSRT-FR) (Derby et al., 2013), delayed recall of story memory and verbal paired associates (Guarch et al., 2008) and visuospatial paired associate learning (Ahmed et al., 2008). Some authors (Dubois and Albert, 2004; Dubois et al., 2007) proposed using memory tests that provide encoding and retrieval facilities, such as the free and cued recall test (Grober and Buschke, 1987; Gainotti et al., 2014) to improve the prediction of MCI-AD conversion. However, a recently review showed inconclusive results (Grober and Buschke, 1987; Carlesimo et al., 2011; Gainotti et al., 2014). It is mandatory to have adequate and well-controlled studies to determine whether that test paradigms can improve the prediction of conversion from MCI to AD.

Thus, most clinical studies assume that memory is a single entity and extrapolate the results of a single memory measure (e.g., delayed recall or recognition task, verbal, or visual memory test) to the global memory functioning. Episodic memory can be subdivided according to the different stages on the reproduction process (e.g., free recall, cued recall, or recognition) and this multicomponent analysis may be used to describe the real risk of MCI to AD conversion. To our knowledge, it is not clear whether measurement of recognition task adds benefit for measuring conversion over time in a clinical cohort.

In the present study, we investigated whether recognition memory tasks in combination with delayed recall measure of episodic memory and CSF biomarkers can predict MCI to AD conversion at 24-month follow-up. We chose to examine this issue using ADNI, a public data set with a large sample and prospective nature. Delayed recall is the most important predictor for MCI to AD conversion. In addition, AD's patient deficits are evident in recognition tasks, with the presence of many false alarms errors. Most studies simplify recognition task by only registering the number of correct identifications (hits), without the inclusion of false alarms in their interpretation. Based on the signal detection theory, a discriminability recognition index (d-prime) can be calculated including both hits and false alarms (Russo et al., 2016). In addition, d-prime has been shown to distinguish healthy older adults from those with a-MCI or mild AD. We hypothesized that the addition of the d-prime measure to the delayed recall task would be good predictor of conversion from a-MCI to AD at 24-month follow-up in combination with CSF biomarkers related to AD, which were significant discriminators in other studies (Tabert et al., 2006; Lanari and Parnetti, 2009; Gomar et al., 2011) and were independently associated with future cognitive decline compared to other surrogate of neurodegeneration as MRI (Vemuri et al., 2009). We also thought that the addition of the traditional recognition score would be not a good predictor in combination with the same markers.

With the concepts explained in these paragraphs, we measured:

- The main effect of the cognitive measures: RAVLT delayed recall, traditional RAVLT recognition, and d-prime.
- The main effect of the known risk factors such as age, sex, education, and APOE genotype.
- The main effect of the CSF biomarkers related to AD: Aβ1-42, P-tau181, and total tau.
- The interaction between RAVLT delayed recall and recognition measures (traditional score and d-prime), which represents the influence of the delayed recall on the magnitude of the recognition task.

Many papers have looked at specific clinical markers and biomarkers in isolation but never set up a study to compare the added effect to predict conversion to dementia. We believed that this analysis would help to interpret the MCI-AD transition in this sample and to propose the d-prime as an early useful cognitive marker to predict AD conversion.

## Materials and methods

### Subjects

Data used in the preparation of this article were obtained from the Alzheimer's disease Neuroimaging Initiative (ADNI) database (adni.loni.usc.edu). The ADNI was launched in 2003 by the National Institute on Aging (NIA), the National Institute of Biomedical Imaging and Bioengineering (NIBIB), the Food and Drug Administration (FDA), private pharmaceutical companies and non-profit organizations, as a $60 million, 5-year public-private partnership. The primary goal of ADNI has been to test whether serial magnetic resonance imaging (MRI), positron emission tomography (PET), other biological markers, and clinical and neuropsychological assessment can be combined to measure the progression of mild cognitive impairment (MCI) and early Alzheimer's disease (AD). Determination of sensitive and specific markers of very early AD progression is intended to aid researchers and clinicians to develop new treatments and monitor their effectiveness, as well as lessen the time and cost of clinical trials.

The Principal Investigator of this initiative is Michael W. Weiner, MD, VA Medical Center, and University of California—San Francisco. ADNI is the result of efforts of many co-investigators from a broad range of academic institutions and private corporations, and subjects have been recruited from over 50 sites across the U.S. and Canada. The initial goal of ADNI was to recruit 800 subjects but ADNI has been followed by ADNI-GO and ADNI-2. To date these three protocols have recruited over 1,500 adults, ages 55–90, to participate in the research, consisting of cognitively normal older individuals, people with early or late MCI, and people with early AD. The follow up duration of each group is specified in the protocols for ADNI-1, ADNI-2, and ADNI-GO. Subjects originally recruited for ADNI-1 and ADNI-GO had the option to be followed in ADNI-2. For up-to-date information, see http://www.adni-info.org (Mueller et al., 2005; Petersen et al., 2010).

A total of 397 subjects with a-MCI from the ADNI study were included in the current analysis. MCI subjects fulfilled criteria for a-MCI (Petersen, 2004): nondemented subjects with memory complaint (global CDR score = 0.5, with a Memory Box score ≥ 0.5), MMSE score of 24–30, a Modified Hachinski Ischemic Score (Rosen et al., 1980) ≤4, a Geriatric Depression Score short form (Sheikh and Yesavage, 1986) <6, and preserved instrumental activities of daily living. Subjects performed at an objective cut-off of 1.5 standard deviations (SD) below education-adjusted cut-off scores on the Logical Memory IIa of the Weschler Memory Scale-Revised (Wechsler, 1987). The summary of the baseline characteristics of the ADNI subjects indicated that the subjects with MCI presented memory-only deficits (Petersen et al., 2010).

The subjects with a-MCI were divided into two groups for the comparison between those who convert to AD dementia during 24-month follow-up and those who did not.

### Psychometric testing

The baseline characteristics of the subjects from ADNI cohort relevant to describe their neuropsychological profile were the Mini Mental State Examination (MMSE) (Folstein et al., 1975); Logical memory test of immediate and delayed recognition, Wechsler Memory Scale III (Wechsler, 1987); Boston Naming Test (Kaplan et al., 1983); Categorical and Phonological Verbal Fluency test (Morris et al., 1989); Digit Span Forward and Backward (Wechsler, 1997); Trail Making Test A and B (Reitan, 1958); GDS short form (Sheikh and Yesavage, 1986); and RAVLT (Rey, 1941, 1964).

The RAVLT consists of five learning trials in which a list of 15 words is read and the subject is asked to immediately recall orally as many items as possible. After an interference list of 15 novel words is read and recalled, subjects are then asked to recall words from the initial list (5-min delayed recall). A 30-min delayed recall trial and recognition test follow. For the recognition test, subjects are presented with a list of the 15 studied words and 15 non-studied foils and are asked to circle all words previously learned.

Discriminability refers to the ability to distinguish target words from distractor words, and is widely considered the best measure of recognition memory accuracy. Discriminability index (d-prime = Z hits rate – Z false alarms rate) is adapted from signal detection theory (Snodgrass and Corwin, 1988) and is analogous to a contrast z score, reflecting the absolute difference in standard deviation units between subject hit rate and false alarm rate (Donaldson, 1992).

### CSF analysis

CSF was available from 198 (49.80%) patients. Methods for cerebrospinal fluid (CSF) acquisition and biomarker measurement using the ADNI cohort has been reported previously (Shaw et al., 2009). In brief, CSF was collected and stored at −80°C at the University of Pennsylvania ADNI Biomarker Core Laboratory. Amyloid-β from peptides 1-42 (Aβ1-42), tau phosphorylated at threonine 181 (P-tau181), and total tau was measured using the multiplex xMAP Luminex platform (Luminex Corp, Austin TX) with Innogenetics (INNOBIA AlzBio3, Ghent, Belgium) immunoassay kit–based reagents.

### APOE ε4 status

APOE ε4 status was considered as a binary variable (i.e., at least one ε4 allele vs. none).

### Statistical analyses

Statistical analysis was performed with SPSS∗ Version 19.0 software for Windows. Independent sample *t*-tests were used to assess differences in clinical, cognitive and biomarkers CSF variables. The χ^2^-test was used for the analysis of gender and APOEε4 carrier status differences between groups. The Pearson's test of correlation was used to measure the degree of relationship between conversion status at 24-month follow-up and the predictive factors. Logistic regression modeling was done to assess the predictive value of all RAVLT measures (Delayed Recall, Recognition, and d-prime), risk factors such as age (in years), sex (male/female), education (in years), APOEε4 carrier status (at least 1 allele vs. none allele, MMSE score, and CSF biomarkers levels for progression to AD. Estimating adjusted odds ratios was used to determine which variables would produce an optimal predictive model, and whether adding tests of interaction between the RAVLT Delayed Recall and RAVLT recognition measures (traditional score and d-prime) would improve prediction of the conversion from a-MCI to AD by including the main effects and two interaction terms (RAVLT Delayed Recall∗RAVLT Recognition; RAVLT Delayed Recall∗d-prime).

## Results

The clinical and demographic characteristics of the subjects as well as CSF biomarkers are listed by conversion status in Table 1. At the 24-month follow-up, 112 (28.2%) subjects developed AD dementia (converters) and 285 (71.8%) subjects did not (nonconverters). Those who developed AD dementia had worse performance on baseline MMSE (*t* = 3.35, *p* = 0.001), Delayed Recall Logical Memory (*t* = 5.17, *p* < 0.001), RAVLT Delayed Recall (*t* = 5.28, *p* < 0.001), RAVLT Recognition (*t* = 3.98, *p* < 0.001), and d-prime (*t* = 3.53, *p* = 0.001), but were not significantly different with respect to sex, age, education, and other neuropsychological tests. The proportion of the individuals with APOEε4 was also significantly higher in those who developed AD dementia than those who did not (χ^2^ = 11.81, *p* = 0.003). CSF Aβ 11-42, tau, and P-tau181 levels were available in 198 subjects from two groups (58 vs. 140) for those who developed AD dementia and those who did not respectively. The group of a-MCI patients who progressed to AD dementia had lower levels of CSF Aβ42 (*t* = 3.71, *p* < 0.001) and CSF AD profile (*t* = 3.78, *p* < 0.001) than the group of non- progressive a-MCI patients.

**Table 1:** **Demographic characteristics, cognitive tests scores, and CSF biomarker profile on the MCI sample at baseline, split according to progression status at 24-month follow-up**.

**Variable**	**a-MCI Non-converters (*n* = 285)**	**a-MCI converters (*n* = 112)**	**Statistical Test t/χ^2^**	***p*-value**
Age, y	74.12 ± 7.1	74.3 ± 6.8	−0.96	0.339
Female, n (%)	97 (34)	44 (39)	0.97	0.352
Education, y	15.7 ± 3.1	15.5 ± 2.9	0.50	0.614
GDS	1.6 ± 1.4	1.4 ± 1.1	1.21	0.226
MMSE	27.2 ± 1.8	26.6 ± 1.6	3.35	0.001
WMS—Immediate Recall	7.5 ± 3.1	6.2 ± 3.1	3.56	0.000
WMS—Delayed Recall	4.2 ± 2.7	2.7 ± 2.2	5.52	0.000
Boston Naming Test	25.7 ± 4.0	24.9 ± 4.2	1.72	0.087
Forward Digit Span	8.2 ± 2.0	8.2 ± 2.0	−0.05	0.961
Backward Digit Span	6.3 ± 2.1	5.9 ± 1.8	1.41	0.159
Category VFT	16.1 ± 5.1	15.1 ± 4.4	1.86	0.064
TMT—A (seconds)	42.9 ± 20.9	49.9 ± 26.3	−2.83	0.395
TMT—B (seconds)	122.6 ± 69.9	152.0 ± 77.7	−3.63	0.000
RAVLT trial 1–5	32.3 ± 9.5	26.7 ± 6.1	5.76	0.000
RAVLT Delayed Recall	3.4 ± 3.5	1.5 ± 2.0	5.28	0.000
RAVLT Delayed-Intrusions	1.4 ± 1.57	1.5 ± 1.9	−0.36	0.721
RAVLT Recognition	10.1 ± 3.4	8.5 ± 3.8	3.98	0.000
RAVLT Recognition-False Alarms	1.9 ± 2.1	2.1 ± 2.6	−0.69	0.487
*d*-prime	1.7 ± 1.0	1.3 ± 0.9	3.42	0.001
APOEε4 carriers, n (%)			11.81	0.003
Noncarriers	147 (51.6)	38 (33.9)		
Carriers 1 allele	111 (38.9)	54 (48.2)		
Carriers 2 alleles	27 (9.5)	20 (17.9)		
CSF biomarkers, pg/ml, n	140	58		
Tau	102.3 ± 66.5	106.6 ± 45.5	−0.45	0.653
A-β1-42	172.7 ± 58.9	141.8 ± 35.2	3.72	0.000
p-Tau 181P	34.2 ± 18.8	38.6 ± 15.7	−1.57	0.117
Tau/ A-β 1-42	0.7 ± 0.7	0.8 ± 0.3	−0.67	0.506
p-Tau 181P/A-β 1-42	0.2 ± 0.2	0.3 ± 0.1	−1.62	0.106
CSF AD profile	0.5 ± 0.2	0.4 ± 0.1	3.78	0.000

*Values are numbers (percentages) or mean ± SD. a-MCI, amnestic mild cognitive impairment; MMSE, Mini-Mental State Examination Test; WMS III, Wechsler Memory Scale III, Logical memory test of immediate and delayed recall; TMT, Trail Making Test A and B; RAVLT, Rey Auditory Verbal Learning Test; CSF, cerebrospinal fluid; AD, Alzheimer's disease*.

Results of logistic regression analyses are shown in Table 2. Of the variables entered into the first step, only RAVLT Delayed Recall and CSF Aβ42 levels reached significance with the odds of 0.79 (95% CI 0.66–0.94) and 0.99 (95% CI 0.98–0.99), respectively. The overall model was statistically significant (χ^2^ = 32.03, *df* = 12, *p* = 0.001). When both interactions (RAVLT Delayed Recall∗RAVLT Recognition; RAVLT Delayed Recall∗d-prime) were entered to evaluate the impact of both recognition measures in predicting conversion, only CSF Aβ42 levels and RAVLT Delayed Recall∗d-prime interaction remained significant in the model with the odds of 0.98 (95% CI 0.97–0.99) and 0.75 (95% CI 0.58–0.97), respectively. Also, the overall model was statistically significant (χ^2^ = 38.23, *df* = 14, *p* < 0.001). The area under the curve was.75, and the percentage of cases classified correctly was 77.4% at *c* = 0.50. Sensitivity was 0.41 and specificity was 0.93 at c = 0.50. The decrease in deviance was the largest for the second step including CSF Aβ42 levels and RAVLT Delayed Recall∗d-prime interaction, indicating that adding d-prime to delayed recall increased the predictive accuracy compared to delayed recall alone (205.354 – 199.149 = 6.205). The drop in deviance was significant (*t* = −52.79, *df* = 196, *p* < 0.001). CSF markers by each potential effect modifier (age, gender, MMSE, RAVLT delayed recall, RAVLT recognition memory, d-prime, or APOE ε4) interaction term was added to model; there was no change in the results.

**Table 2:** **Results of logistic regression analysis**.

**Variable**	**Beta**	***SE***	**Wald**	**Df**	***p*-value**	**OR (95% CI)**
Age	0.022	0.025	0.777	1	0.38	1.02 (0.97–1.07)
Gender (Male)	0.013	0.389	0.001	1	0.97	1.01 (0.47–2.17)
Education	−0.113	0.059	3.631	1	0.06	0.89 (0.79–1.00)
MMSE	−0.150	0.101	2.212	1	0.14	0.86 (0.71–1.05)
RAVLT Delayed Recall	−0.239	0.092	6.764	1	**0.01**	0.79 (0.66–0.94)
RAVLT Recognition	−0.093	0.281	0.110	1	0.74	0.91 (0.52–1.58)
*d*-prime	0.379	0.705	0.288	1	0.59	1.46 (0.37–5.82)
APOEε4	−0.066	0.378	0.030	1	0.86	0.94 (0.45–1.96)
Tau	−0.009	0.005	3.524	1	0.06	0.99 (0.98–1.00)
Aβ_1−42_	−0.014	0.005	9.302	1	**0.00**	0.99 (0.98–0.99)
P-tau_181_	0.012	0.016	0.636	1	0.42	1.01 (0.98–1.04)
RAVLT Delayed Recall*RAVLT Recognition	0.036	0.035	1.058	1	0.30	1.04 (0.97–1.11)
RAVLT Delayed Recall*d-prime	−0.283	0.132	4.596	1	**0.03**	0.75 (0.58–0.98)

*MMSE, Mini-Mental State Examination Test; RAVLT, Rey Auditory Verbal Learning Test; APOE, Apolipoprotein E; Aβ1-42, Amyloid-β from peptides 1-42; P-tau181, tau phosphorylated at threonine 181; Standard error; DF, Degrees of freedom; OR, Odds ratio; CI, Confidence interval*.

There were weak positive correlations between conversion ratio to AD dementia and MMSE, RAVLT Delayed Recall, RAVLT Recognition, and d-prime (*r* = 0.207, 0.299, −0.215, 0.254, respectively, *n* = 397, *p* < 0.05) (Table 3). There was also a significant but negative correlation between conversion ratio to AD dementia and CSF Aβ42 levels (*r* = −0.659, *n* = 198, *p* < 0.001). There were also a significant but positive correlation between conversion ratio to AD dementia and APOEε4 carrier status, (*r* = 0.355, *n* = 397, *p* < 0.001), and CSF P-tau181 levels (*r* = 0.429, *n* = 198, *p* < 0.001).

**Table 3:** **Correlations between conversion status at 24-month follow-up and the predictive factors**.

	**Conversion ratio**	**MMSE**	**RAVLT Delayed Recall**	**RAVLT Recognition**	***d*-prime**
Conversion ratio	1.000	0.207^*^	0.299^*^	0.215^*^	0.254^*^
MMSE	0.207^*^	1.000	0.515^*^	0.457^*^	0.517^*^
RAVLT Delayed Recall	0.299^*^	0.515^*^	1.000	0.622^*^	0.715^*^
RAVLT Recognition	0.215^*^	0.457^*^	0.622^*^	1.000	0.876^*^
*d*-prime	0.254^*^	0.517^*^	0.715^*^	0.876^*^	1.000

*Values are Pearson's Correlation Coefficient (r). MMSE, Mini-Mental State Examination Test; RAVLT, Rey Auditory Verbal Learning Test*.

* *p < 0.05*.

## Discussion

In this study, we demonstrated that baseline CSF Aβ1-42 levels, RAVLT Delayed Recall, and the combination of RAVLT Delayed Recall and Recognition Discriminability Index (d-prime) were the strongest predictors of conversion from a-MCI to AD at 24-month follow-up in patients in the ADNI sample. These results were independent of other factors known to increase the risk of developing dementia, such as age, gender, education level, and APOEε4 status (Tabert et al., 2006; Lanari and Parnetti, 2009; Gomar et al., 2011). We also showed that the combination of RAVLT Delayed Recall and traditional recognition measure may not predict MCI-AD conversion.

One might argue that the use of verbal episodic memory markers in this context is redundant. However, this may not be correct because the RAVLT memory measures examined as a predictor in this paper was not part of the study inclusion criteria in ADNI cohort (WMS-R logical memory was used). Nevertheless, evaluation of different components of verbal episodic memory deficit as a predictor in patients with a-MCI may be an advantage to interpret our patients in clinical practice or to improve their recruitment in clinical trials. Other important issue is that the diagnostic conversions over the 24-month period should be viewed with some caution. A 24-month change period is not a sufficient amount of time to draw conclusions regarding the likelihood of clinical change. However, we preferred not include the 36- or 48-month period due to partially missing baseline data.

Numerous studies that characterized the episodic memory deficit in AD have used word list learning tasks such as those from the Consortium to Establish a Registry for Alzheimer Disease (CERAD) (Welsh et al., 1992), the RAVLT (Rey, 1941, 1964), and the California Verbal Learning Test (CVLT) (Delis et al., 1991). These studies consistently showed that AD patients are equally impaired (relative to age-matched controls) on recognition and free recall components of the tasks. This pattern of performance is consistent with impaired consolidation rather than ineffective retrieval of new information (Delis et al., 1991). This memory profile is a useful measure for a clinical diagnosis and classification MCI subjects.

However, MCI in general and a-MCI in particular are heterogeneous clinical constructs, and the reported literature that investigated the role of different verbal episodic memory task as a predictor of cognitive decline in MCI is variable, with inhomogeneous results. Explicit measures of delayed recall have been reported the best predictors of the development of AD in MCI subjects (Gainotti et al., 2014). With respect to the recognition memory task, we found few studies that assessed their predictive value. Rabin et al. (2009) revealed that logical memory recognition best predicted the progression to AD followed closely by the delayed recall condition of CVLT-II. Yang et al. (2012) subdivided a-MCI sample from ADNI cohort into two subtypes, encoding failure (a-MCI-E) vs. retrieval deficit (a-MCI-R) but did not find that a-MCI-E was an independent prognostic factor to predict the progression to AD. In addition to the neuropsychological predictors, current criteria and recommendations (Dubois et al., 2007; Albert et al., 2011) proposed that incorporation of distinctive and reliable biomarkers of underlying AD to predict MCI-AD conversion. Most prior studies of CSF biomarkers have showed only modest prognostic accuracy for prediction of conversion from MCI to AD (Vemuri et al., 2009). On the other hand, most of these studies aimed to identify the predictive value of a set of commonly measured variables, with classification accuracies reaching up to 80%. However, none of these prior studies have investigated the predictive value when combining different related variables to homogenize the group of subjects with higher risk of conversion.

We have found that delayed recall can make important influence to recognition task in patients who convert to dementia, when the hits and false alarms are considered. Considering that memory is not a unitary concept, the strategy of combining recall and recognition memory measures might provide a more accurate assessment of MCI individuals who perform poorly on list learning tasks or allow to develop an empirical perspective of subtyping within a-MCI that may identify more homogenous subgroups reflecting common etiology and better predictors of decline. We considered that the simultaneous analysis of CSF biomarkers or the combination of different assessment yielded a better prediction of conversion from MCI to AD.

Future research should more closely examine which memory processes are tapped by specific tasks and the nature and extent of decline in these processes during the insidious transition from healthy aging to MCI to AD. This suggested subdivision within a-MCI based on recall and recognition performance need to be assessed using traditional delayed recall and recognition measures, but with inclusion of additional variables (e.g. d-prime). We also expect that future prediction studies consider the co-occurrence effects of different variables (interactions), and not only traditional additive (main effects) models.

## Ethics statement

The study was approved by the Institute of Neurological Research FLENI Ethics and Research Committee. The paper was reviewed and was acceptable for submission by the Alzheimer's Disease Neuroimaging Initiative Data and Publications Committee (ADNI DPC).

## Author contributions

All authors contributed extensively to the work presented in this paper. MR designed the study. JC and SV helped to evaluate and edit the manuscript. GS helped in the interpretation of data related to biomarkers. RA supervised development or research work and helped in data interpretation and manuscript evaluation.

### Conflict of interest statement

The authors declare that the research was conducted in the absence of any commercial or financial relationships that could be construed as a potential conflict of interest.
